# Supplementary material for: Modelling the impact of interventions on imported, introduced and indigenous malaria infections in Zanzibar, Tanzania
Source: Nat Commun. 2023 May 12;14:2750. doi: 10.1038/s41467-023-38379-8 (PMC10182017; doi:10.1038/s41467-023-38379-8)
Supplement: Supplementary file 1 — Supplementary Information [file 41467_2023_38379_MOESM1_ESM.pdf]

Supplementary Information

Modelling the impact of interventions on imported, introduced and indigenous malaria cases in Zanzibar, Tanzania

Aatreyee M. Das[1,2]*, Manuel W. Hetzel[1,2], Joshua O. Yukich[3], Logan Stuck[3]†, Bakar S. Fakih[1,2,4], Abdul-wahid H. Al-mafazy[5]‡, Abdullah Ali[5], Nakul Chitnis[1,2]

1 Swiss Tropical and Public Health Institute, Allschwil, Switzerland
2 University of Basel, Basel, Switzerland
3 Center for Applied Malaria Research and Evaluation, Department of Tropical Medicine, Tulane University School of Public Health and Tropical Medicine, New Orleans, LA, USA
4 Ifakara Health Institute, Dar es Salaam, United Republic of Tanzania
5 Zanzibar Malaria Elimination Programme, Zanzibar, United Republic of Tanzania

S1 Methods

S1.1 Modelling of interventions

S1.1.1 RCD at a range of levels of follow up

The percentage of malaria cases diagnosed at a health facility that are followed up by a District Malaria Surveillance Officer is given by η. η is varied between 0% and 100% to model the extreme values of removing RCD altogether, and perfect follow up of all cases diagnosed at a health facility.

S1.1.2 RCD at a range of levels of treatment seeking

The rate at which people seek treatment is 2.9×10^{-4} per day in Pemba and 6.1×10^{-4} per day in Unguja. This was calculated by considering the median number of malaria infections diagnosed at a health facility per month per district on Pemba and Unguja, and scaling by the number of districts and 30 days in a month [1]. This was increased by a factor of 2 or 3 to simulate increase in treatment as an intervention.

S1.1.3 RCD with follow up of neighbours

Currently, neighbours are not included in RCD. We simulated the testing and treatment of 20 and 100 neighbours, as well as the index household. From the RADZEC study data, we estimated

*Corresponding author
†Current affiliations: Amsterdam Institute for Global Health and Development, Amsterdam, Netherlands and Amsterdam University Medical Centers, Amsterdam, Netherlands
‡Current affiliation: RTI International, Dar es Salaam, United Republic of Tanzania

that the targeting ratio amongst neighbouring households is around 0.7 (95% confidence interval (CI): 0.4–1.3) in Pemba and 1.3 (95% CI: 0.9–1.9) in Unguja.

Thus, the RCD term was modified to

$$\varphi_k = \rho I_k(\tau_k^{(h)}\nu_k^{(h)} + \tau_k^{(n)}\nu_k^{(n)})\eta\xi_k, \tag{1}$$

with $\nu_k^{(n)}$ either 20 or 100.

S1.1.4 Switching from RCD to RDA

When modelling RDA, we considered that all index household members and neighbours (when included) would receive treatment regardless of disease status. Thus, the diagnostic test sensitivity, ρ, was changed from 34% to 100%.

S1.1.5 Treatment of a proportion of cases brought on to Zanzibar by travelling humans (either residents or visitors)

Currently, prophylaxis is not provided to travellers to mainland Tanzania. Similarly, there is no screen-and-treat programme for entrants to Zanzibar. We include treatment of imported cases as a potential intervention in our model, in order to evaluate what proportion of cases must be treated to achieve different reductions in prevalence on Pemba and Unguja [2]. We modify Eq. (4) in the main text to have a θ^{outbound}, which includes treatment for visitors from mainland Tanzania on their outbound journey to Zanzibar, and θ^{return} for Zanzibari residents that receive treatment on their return journey to Zanzibar. Thus, the base form of the equation becomes

$$\frac{\mathrm{d}I_k}{\mathrm{d}t} = \sum_{i=1}^{3}\left(\beta_i\left(\frac{\sum_{j=1}^{3} N_j\theta_{ij}^{\text{outbound}} I_j}{\sum_{j=1}^{3} N_j\theta_{ij}}\right)\theta_{ik}^{\text{return}}\right)(1-I_k) - (\mu+\varphi_k)I_k, \tag{2}$$

where

$$\theta^{\text{outbound}} = \begin{pmatrix} 0.991 & 0.004 & (1-O)*5.7\times10^{-5} \\ 0.003 & 0.970 & (1-O)*5.3\times10^{-4} \\ 0.006 & 0.026 & 0.999 \end{pmatrix}, \tag{3}$$

and

$$\theta^{\text{return}} = \begin{pmatrix} 0.991 & 0.004 & 5.7\times10^{-5} \\ 0.003 & 0.970 & 5.3\times10^{-4} \\ (1-R)*0.006 & (1-R)*0.026 & 0.999 \end{pmatrix}. \tag{4}$$

O represents the proportion of travellers from mainland Tanzania receiving treatment such that they are no longer infected upon entering Zanzibar, and R represents the proportion of Zanzibari residents receiving treatment such that they are no longer infected upon returning to Zanzibar. We always simulate equal proportions of outbound and return cases being treated (i.e. $O = R$)

S1.1.6 Reductions in the malaria transmission rate

The rate at which malaria is transmitted from one human to another can be reduced through vector control interventions such as the use of long-lasting insecticidal nets, indoor residual

	Das et al (2022)		
	**Baseline**	**No RCD**	**Percentage change**
**Pemba**	5,549	6,121	10.3%
**Unguja**	10,538	11,385	8.0%
	**New model**		
	**Baseline**	**No RCD**	**Percentage change**
**Pemba**	5,554	6,141	10.6%
**Unguja**	10,533	11,375	8.0%

Table S1: Median number of infected individuals at equilibrium out of 500 stochastic simulations using the simpler version of the model presented in Das *et al* (2022), as compared to the current model. The results for baseline, the counterfactual of no reactive case detection, and the percentage increase in malaria prevalence is shown.

spraying, and larval source management. As we do not explicitly model mosquitoes or the vectorial capacity, we reduce the transmission parameter, β, to simulate increases in vector control.

Thus, for this intervention, β is replaced by $\beta(1-r)$ where r is the reduction in vectorial capacity. As vectorial capacity is proportional to the number of susceptible humans infected by an infected human per day, β is proportional to the vectorial capacity, and any reduction in β could arise from a proportional reduction in the vectorial capacity [3]. Values ranging from 0.25 to 0.9 were tested for r on Pemba and Unguja, and values ranging from 0.1 to 0.3 for r on mainland Tanzania.

S2 Results

S2.1 Comparison of models

The current model was compared to the simpler version of the model presented in Das *et al* (2022) [2]. The counterfactual increase in malaria prevalence expected from stopping RCD was found to be very similar in both cases (Table S1). The slight difference is due to the stochastic nature of the model implementation.

S2.2 Comparison of interventions

The impact of each intervention alone was tested by changing one factor at a time and plotting the final equilibrium reached 40 years after the introduction of the intervention. All other factors were held at their baseline value, given in Table 3 in the main text. The results from this analysis are shown in Fig S1. Most intervention parameters had an approximately linear relationship with malaria incidence, but the relationship between the percentage of travellers treated and the incidence of infections was mildly concave, and the relationship between a reduction in the malaria transmission rate in Zanzibar and the incidence of infections was steeply curved. This suggests that even small increases in vector control may have a disproportionately large impact with regard to reducing malaria incidence on Zanzibar.

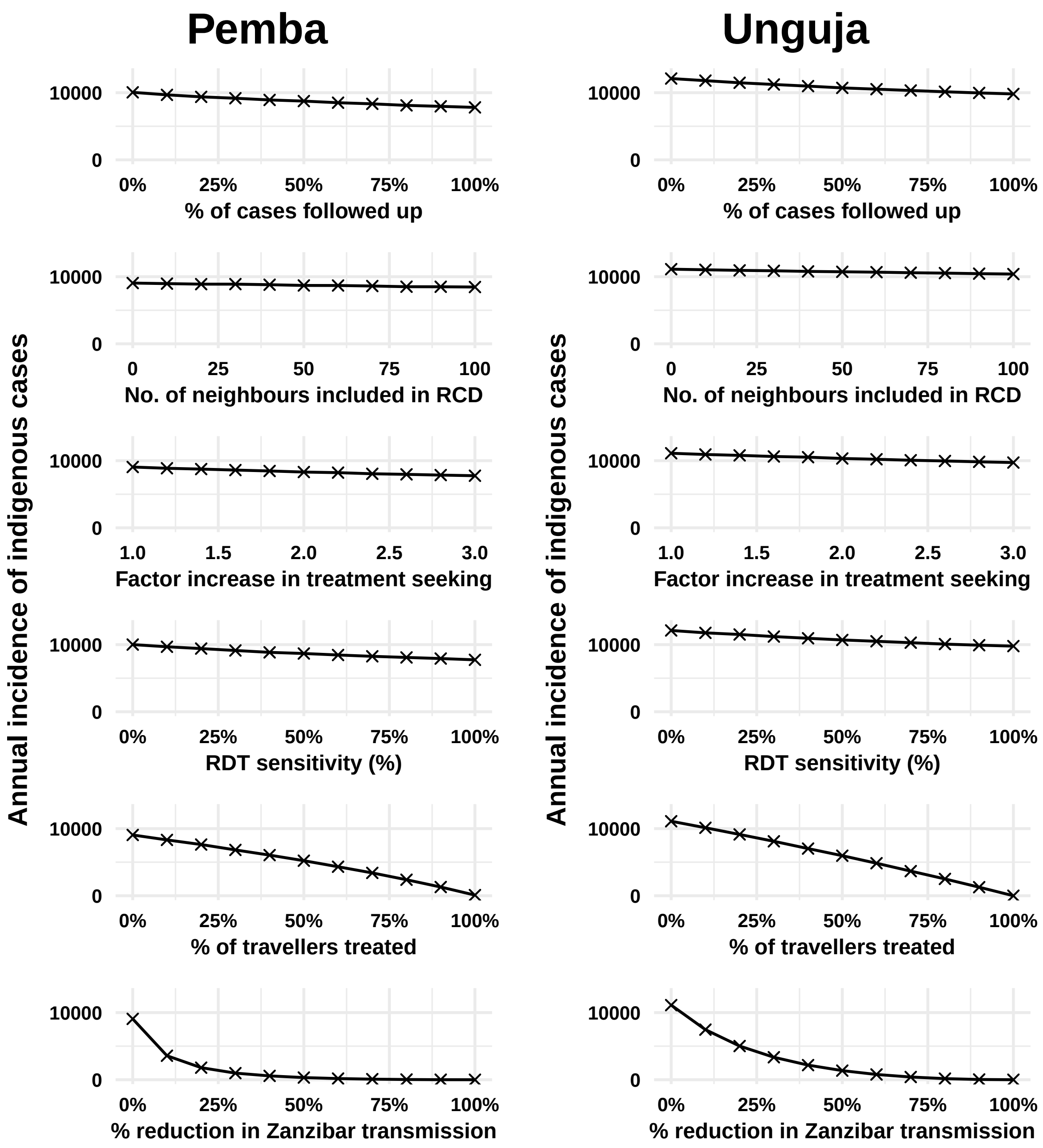

Figure S1: Median yearly incidence of indigenous cases out of 500 simulations in the 40th year after the start of each intervention. At each point, only the parameter on the x-axis has been changed, with all other parameters remaining at the baseline value. RCD: reactive case detection; RDT: rapid diagnostic test.

S2.3 Impact of parameter uncertainty

Parameter uncertainty was considered in the same way as described in Das *et al* (2022) [2]. Simulations were run with a range of parameter values based on the uncertainty in the data, taking the posterior distribution when an uninformative prior is updated with the observed data. The parameters varied and the distributions from which they were sampled were as follows:

- The equilibrium malaria prevalence on Pemba, $I_1^* \sim \text{Beta}(32, 2242)$;
- The equilibrium malaria prevalence on Unguja, $I_2^* \sim \text{Beta}(92, 3196)$;

- The targeting ratio in index households in Pemba, $\tau_1^{(h)} \sim \frac{\text{Beta}(20,427)}{I_1^*}$;
- The targeting ratio in index households in Unguja, $\tau_2^{(h)} \sim \frac{\text{Beta}(64,470)}{I_2^*}$;
- The targeting ratio in neighbouring households in Pemba, $\tau_1^{(n)} \sim \frac{\text{Beta}(13,1147)}{I_1^*}$;
- The targeting ratio in neighbouring households in Unguja, $\tau_2^{(n)} \sim \frac{\text{Beta}(26,1619)}{I_2^*}$;
- The number of people tested by the RCD programme in the index household in Pemba, $\nu_1^{(h)} \sim \text{Normal}(7.02, 0.24)$;
- The number of people tested by the RCD programme in the index household in Unguja, $\nu_2^{(h)} \sim \text{Normal}(6.36, 0.25)$;
- The number of people tested by the RCD programme in neighbouring households in Pemba, $\nu_1^{(n)} \sim \text{Normal}(20.36, 0.50)$;
- The number of people tested by the RCD programme in neighbouring households in Unguja, $\nu_2^{(n)} \sim \text{Normal}(18.76, 0.58)$.

Subscripts of *1* and *2* indicate Pemba and Unguja, respectively. Parameter values with the 95% interval values can be found in Table S2.

**Variable or parameter**	**Mean values [95% CI]**			**Source**
	Pemba	Unguja	Mainland	
I_k^*	1.36% [0.96-1.93]	1.18% [0.86-1.61]	7.79%	[4, 5]
N_k	406,848	896,721	43,625,354	[6]
θ_{ij}	$\begin{array}{c|ccc} & \text{Pemba} & \text{Unguja} & \text{Mainland} \\ \text{Pemba} & 0.991 & 0.004 & 5.7\times10^{-5} \\ \text{Unguja} & 0.003 & 0.970 & 5.3\times10^{-4} \\ \text{Mainland} & 0.006 & 0.026 & 0.999 \end{array}$			[4]
μ	0.005 day^{-1}	0.005 day^{-1}	0.005 day^{-1}	[7, 8]
$\tau_k^{(h)}$	3.2 [2.0-4.8]	10.0 [8.0-12.6]	N/A	[4]
$\tau_k^{(n)}$	0.7 [0.4-1.3]	1.3 [0.9-1.9]	N/A	[4]
$\nu_k^{(h)}$	7.0 [6.5-7.5]	6.3 [5.9-6.9]	N/A	[4]
$\nu_k^{(n)}$	20.4 [19.4-21.4]	18.8 [17.6-19.9]	N/A	[4]
ρ^*	34%	34%	N/A	[4]
η^*	35.3%	35.3%	N/A	[1]
ξ^*	2.9×10^{-4} day^{-1}	6.1×10^{-4} day^{-1}	N/A	[4, 1]

Table S2: Variable and parameter values at baseline and sources. Where a range of parameter values was tested in the uncertainty analysis, the 95% confidence interval for the range of values tested is given. The superscripts (h) and (n) indicate the index household and neighbouring households, respectively.

100 random values were selected from these parameter distributions, and each set of values was simulated with five different seeds, forming a total of 500 simulations for each intervention

scenario. The final equilibrium value reached for a range of interventions, along with the uncertainty stemming from both the parameter and stochastic variation, is shown in Fig S2a. The distribution of annual incidence at equilibrium can be seen in Fig S2b. The impact of parameter uncertainty on the probability of reaching elimination was also examined and found to be minor (see Fig S3). Even when parameter uncertainty is included, elimination is only observed when there is 100% importation treatment in the absence of transmission reduction interventions.

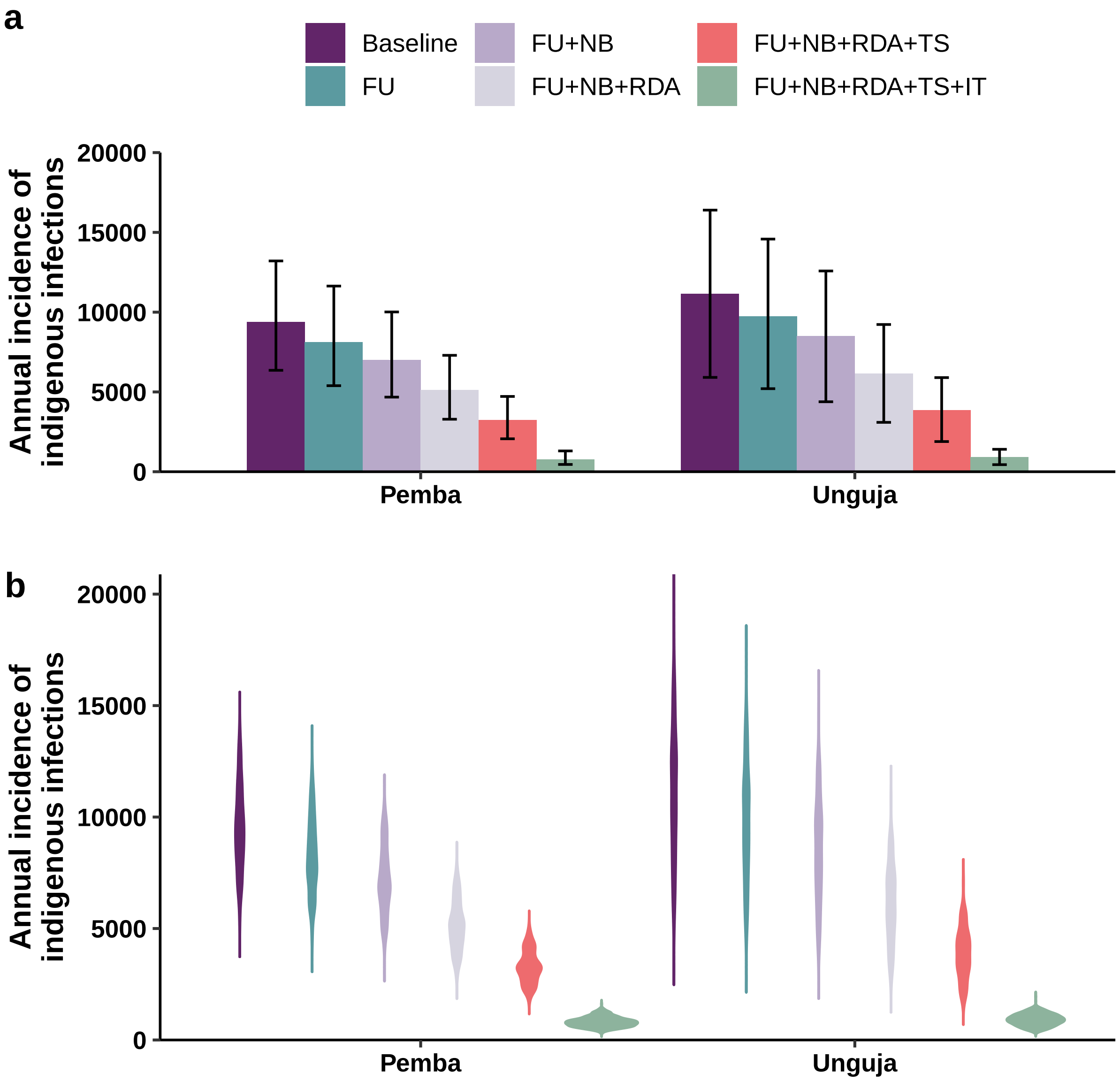

Figure S2: Yearly incidence of indigenous infections 40 years after the start of interventions (i.e. once equilibrium is reached) **a)** Bar chart showing the median, 2.5th and 97.5th percentiles out of n=500 simulations that incorporate both stochastic and parameter uncertainty. **b)** Violin plot of the annual incidence of indigenous infections in the 40th year after the start of interventions. FU: 100% follow up; NB: 100 neighbours included in testing and treatment; RDA: reactive drug administration; TS: three times the baseline treatment seeking rate; IT: treatment of 90% of travellers arriving on Zanzibar.

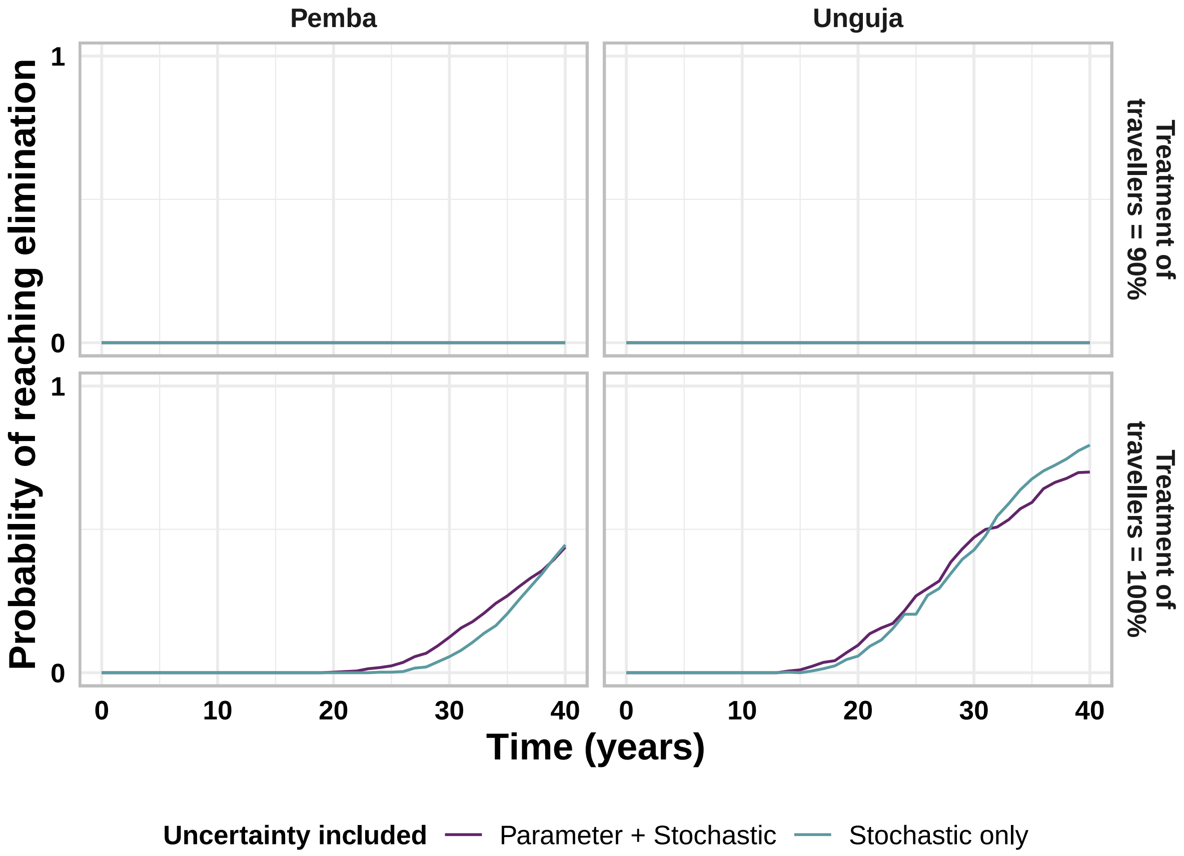

Figure S3: Proportion of stochastic simulations reaching elimination (three years with zero indigenous cases), starting from the introduction of all RCD-related interventions and treatment of imported cases, comparing stochastic uncertainty to the combination of parameter and stochastic uncertainty.

S2.4 Sensitivity analysis

A sensitivity analysis was conducted using the Sobol method to characterise the impact of parameter variation on model outputs. 32,768 parameter values were sampled from uniform distributions for each parameter using Saltelli sampling [9, 10]. The bounds of the uniform distributions corresponded to 95% confidence intervals found in the literature or, when such bounds were not available, the point estimate of the parameter ±50%. Upper and lower bounds and data sources can be found in Table S3. The upper bound of the proportion of time spent by mainland Tanzania residents on Pemba and Unguja was calculated by scaling the upper bound from Le Menach *et al* (2011) by the proportion of people residing on mainland Tanzania as compared to Pemba or Unguja. The model was then run using these parameter sets and a different seed for each parameter set, and the outputs were used to calculate Sobol indices. First order and total Sobol indices were calculated using the SALib package (version 1.4.5) in Python [10].

This analysis suggests that the main outputs of malaria prevalence and the annual incidence of indigenous infections are most sensitive to the estimates of the transmission parameter (Fig S4 and S5). As these are back-calculated from the baseline malaria prevalence, the need for accurate estimates of the prevalence in the general population is important for a correct estimate of the effective human-to-human malaria transmission rate. An accurate estimate of the baseline prevalence is also key to estimating the probability of elimination being reached under different

Parameter	Point estimate	Lower bound	Upper bound	Reference
Transmission rate on Pemba (β_{Pemba})	0.0048	0.0024	0.0072	±50%
Transmission rate on Unguja (β_{Unguja})	0.0037	0.0019	0.0056	±50%
Transmission rate on mainland Tanzania (β_{Mainland})	0.0054	0.0027	0.0081	±50%
Mean duration of infection ($1/\mu$)	200	184	237	[11]
Targeting ratio on Pemba ($\tau^{(h)}_{\text{Pemba}}$)	3.1	2.0	4.8	RADZEC data ([4])
Targeting ratio on Unguja ($\tau^{(h)}_{\text{Unguja}}$)	10.1	8.0	12.7	RADZEC data ([4])
Treatment seeking rate on Pemba (ξ_{Pemba})	2.9×10^{-4}	1.5×10^{-4}	4.4×10^{-4}	±50%
Treatment seeking rate on Unguja (ξ_{Unguja})	6.1×10^{-4}	3.1×10^{-4}	9.2×10^{-4}	±50%
RDT sensitivity (ρ)	34%	0%	100%	Full range
Follow up of index cases (η)	35%	0%	100%	Full range
Movement from Pemba to Unguja (θ_{UP})	0.0032	0.0016	0.0048	±50%
Movement from Pemba to mainland Tanzania (θ_{MP})	0.0061	0	0.12	[12]
Movement from Unguja to Pemba (θ_{PU})	0.0039	0.0019	0.0058	±50%
Movement from Unguja to mainland Tanzania (θ_{MU})	0.026	0	0.12	[12]
Movement from mainland Tanzania to Pemba (θ_{PM})	5.7×10^{-5}	0	0.0011	[12]
Movement from mainland Tanzania to Unguja (θ_{UM})	5.3×10^{-4}	0	0.0025	[12]

Table S3: Parameter bounds for sensitivity analysis.

intervention conditions.

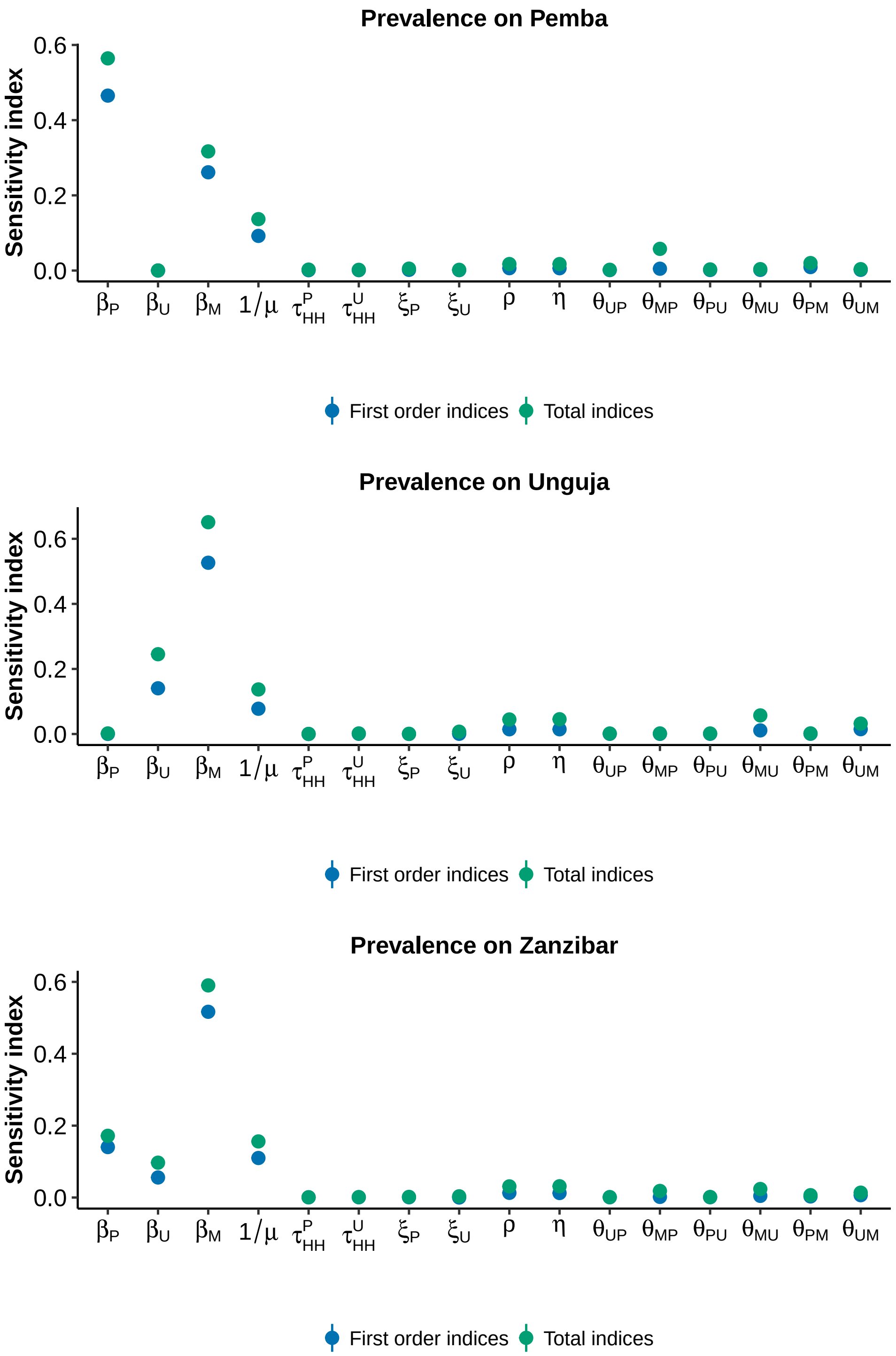

Figure S4: First order and total Sobol indices for each parameter tested when the overall malaria prevalence is considered as the output. Note, the 95% confidence intervals are smaller than the point sizes and so are not visible. The model output of malaria prevalence on Zanzibar as a whole was calculated by multiplying the expected prevalence on each island by the population size of each island, summing to get the total number of infected people on both islands, and then dividing by the summed population across both islands.

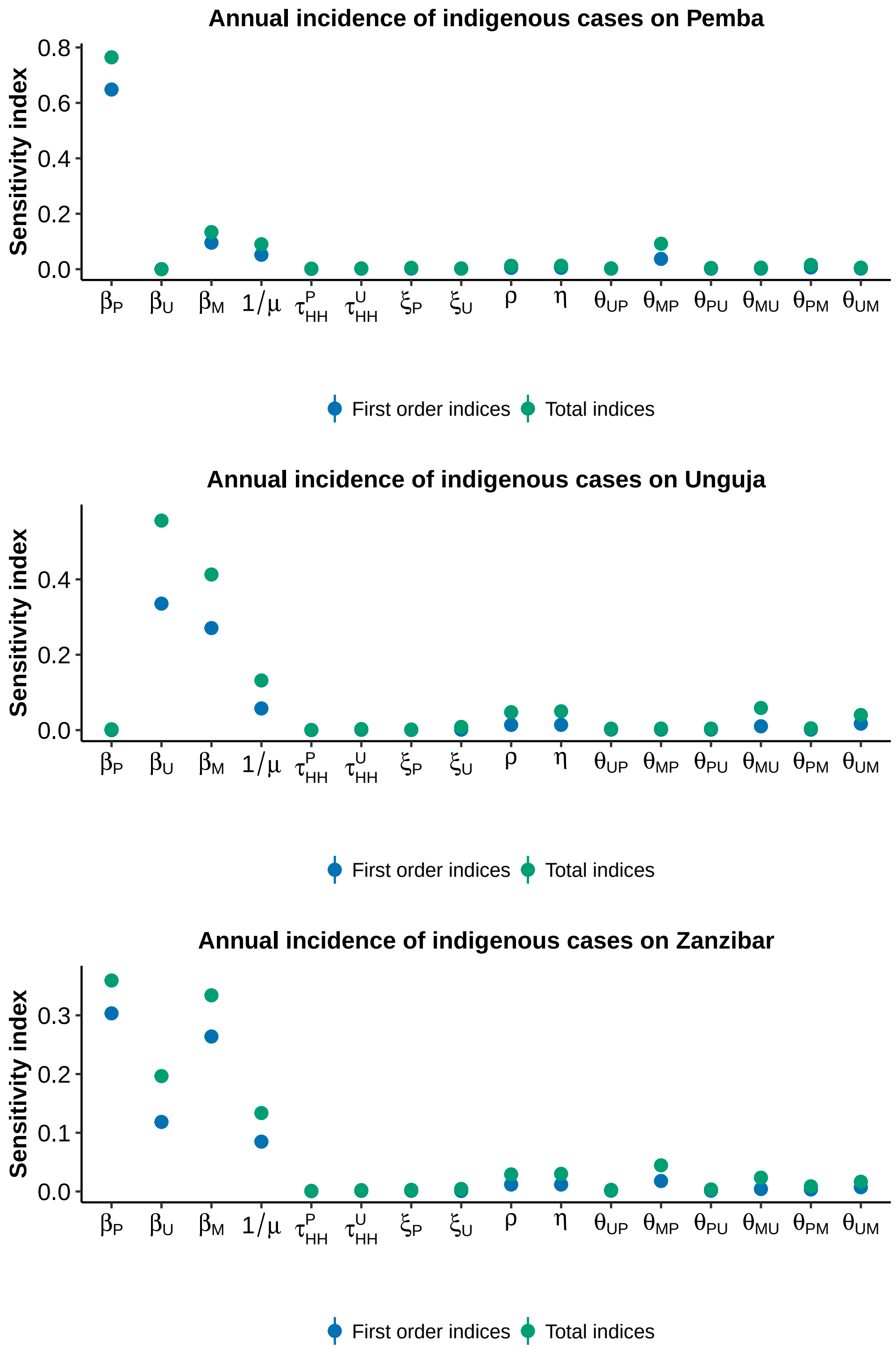

Figure S5: First order and total Sobol indices for each parameter tested when the annual incidence of indigenous cases is considered as the output. Note, the 95% confidence intervals are smaller than the point sizes and so are not visible. The total incidence of indigenous cases for Zanzibar as a whole was calculated by summing the incidence of indigenous cases on each island.

S2.5 The impact of a fixed versus a varying targeting ratio

The targeting ratio is calculated from the RADZEC study data and is assumed to be fixed in the main text, regardless of the population malaria prevalence. This implies that cases do not become more clustered as the disease prevalence falls. In comparison, Chitnis *et al* (2019) consider a targeting ratio that varies depending on prevalence and the number of people tested, with the ratio of malaria infections amongst those tested as compared to the general population decreasing as prevalence and the number of people tested increases [13]. The following function was found to best estimate the targeting ratio, τ, for geo-located prevalence data collected in Zambia:

$$\tau(\nu, I) = \exp\left(\left(-\alpha_1 \ln(I) + \frac{\alpha_2}{\nu} - \frac{\alpha_3}{\nu}\ln(I)\right)\frac{N-\nu}{N}\right) \mid \tau(\nu, I) \geq 1, \tag{5}$$

where τ is the targeting ratio, ν is the number of people tested, not including the index case, N is the total population, and α_1, α_2 and α_3 are fitted parameters with values $\alpha_1 = 0.23$ (95% credible interval (CI): 0.16, 0.29); $\alpha_2 = -1.40$ (CI: -2.77, -0.02) and $\alpha_3 = 2.87$ (CI: 1.13, 4.59) [13].

In order to compare running the model with a fixed targeting ratio and a varying targeting ratio, we take the function from Chitnis *et al* (2019) that is fitted to data from Zambia, and apply a scaling factor to adjust the targeting ratio so that the targeting ratio matches between Eq. (5) and the targeting ratio for the index household in the RADZEC data ($\tau^{(h)}$ in the main text). Thus, the equation we used to generate a varying τ was given by

$$\tau(\nu, I) = A\exp\left(\left(-\alpha_1 \ln(I) + \frac{\alpha_2}{\nu} - \frac{\alpha_3}{\nu}\ln(I)\right)\frac{N-\nu}{N}\right) \mid \tau(\nu, I) \geq 1, \tag{6}$$

where A was calculated to be 0.19 for Pemba and 0.42 for Unguja, in order to match the targeting ratios calculated by Eq. (6) and the targeting ratio seen in the RADZEC study data. Thus, as the malaria prevalence decreases due to the introduction of new interventions, the targeting ratio increases and the effectiveness of RCD increases.

Running the model with a varying τ and a fixed τ, we see that the difference in the targeting ratio is not substantial even when considering the maximum RCD interventions tested i.e. RDA with triple the usual treatment seeking rate and 100 neighbours included in treatment (see Fig S6). These interventions maximise the effect of the targeting ratio and so are the ones where we'd expect to see the largest difference between the blue and purple lines in Fig S6. When RCD finds and treats a lot of cases, a targeting ratio that improves as the prevalence falls can provide an optimistic outlook of potentially eliminating malaria earlier than when considering a fixed targeting ratio, which makes the more conservative assumption of no increase in case clustering as prevalence decreases. Nonetheless, given the difference is small, we have used a fixed targeting ratio for all simulations shown in the main text.

S2.6 The impact of varying the definition of malaria re-establishment

Currently, we consider a simulation to have reached elimination when three years have passed with zero incidence of indigenous cases. However, if an indigenous case appears after this three year period, we count this as malaria re-establishment and thus losing 'eliminated' status. In contrast, the World Health Organization defines the minimum indication of re-establishment of transmission as *'the occurrence of three or more indigenous malaria cases of the same species per year in the same focus, for three consecutive years'* [14]. Since no country that has been certified as malaria-free has lost certification, we additionally modelled the impact of assuming that once a region eliminates malaria, it stays malaria-free. Out of the 500 simulations, when a simulation reaches three years with zero incidence of indigenous cases, we assume it remains

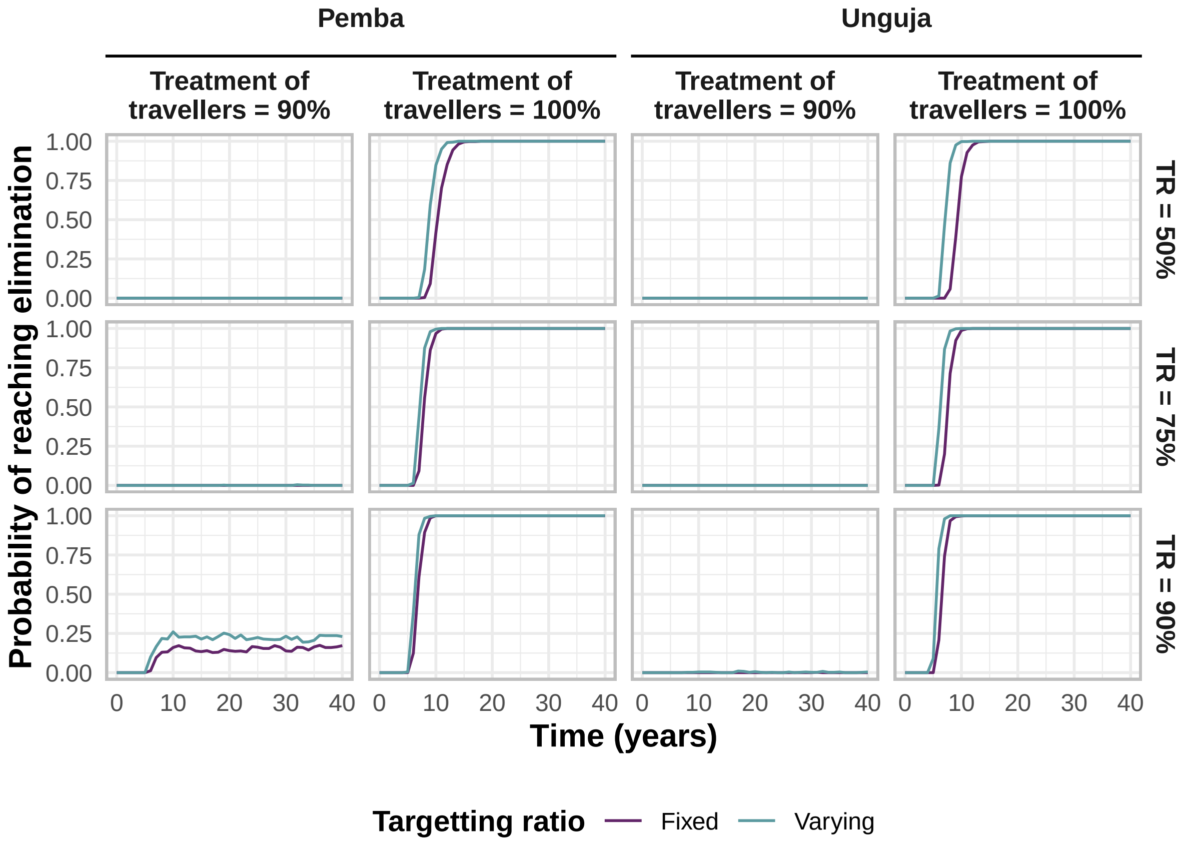

Figure S6: Proportion of stochastic simulations reaching elimination (three years with zero indigenous cases), starting from the introduction of all reactive case detection-related interventions, comparing a more conservative definition of the targeting ratio, where the ratio is constant regardless of prevalence, and a definition where the targeting ratio increases as the malaria prevalence decreases. TR: transmission reduction.

at zero indigenous cases indefinitely into the future. The probability of reaching elimination is shown in Fig S7, with the assumption of remaining malaria-free after elimination labelled as ‘cumulative’ and the more strict definition of malaria re-establishment (losing ‘eliminated’ status after the appearance of one indigenous case) labelled as ‘transient’. We observe that in the majority of cases, the definition of re-establishment does not impact the proportion of runs reaching elimination. Only in the case where the number of indigenous cases is typically zero, but not always (90% importation treatment with 90% reduction in the transmission rate on Pemba) does the definition of re-establishment make a substantial difference to the number of runs reaching elimination.

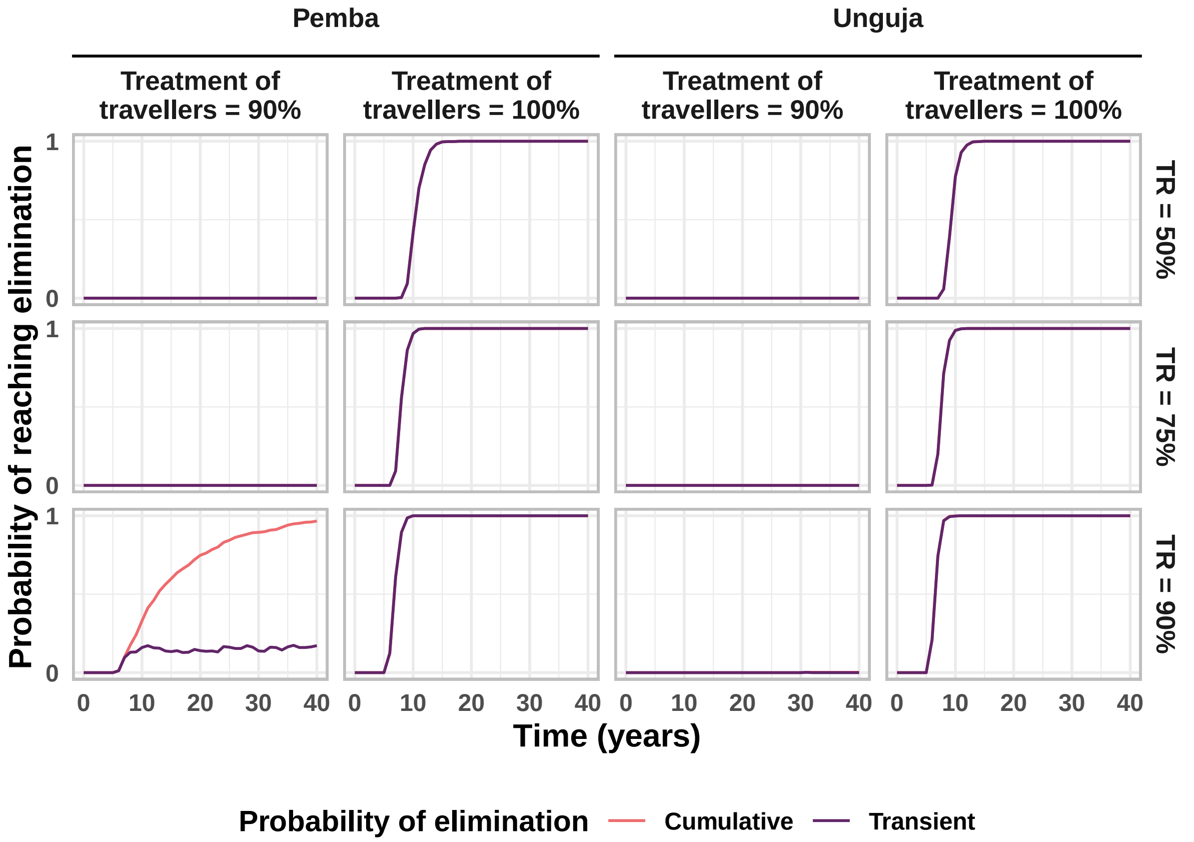

Figure S7: Proportion of stochastic simulations reaching elimination (three years with zero indigenous cases), starting from the introduction of all reactive case detection-related interventions and treatment of imported cases, comparing a transient probability of elimination (where 'eliminated' status is lost after the appearance of one indigenous case), to a cumulative probability of elimination (once a simulation reaches elimination, it stays malaria-free with zero indigenous cases). TR: transmission reduction.

S3 Additional figures

S3.1 Time-series plots for individual interventions

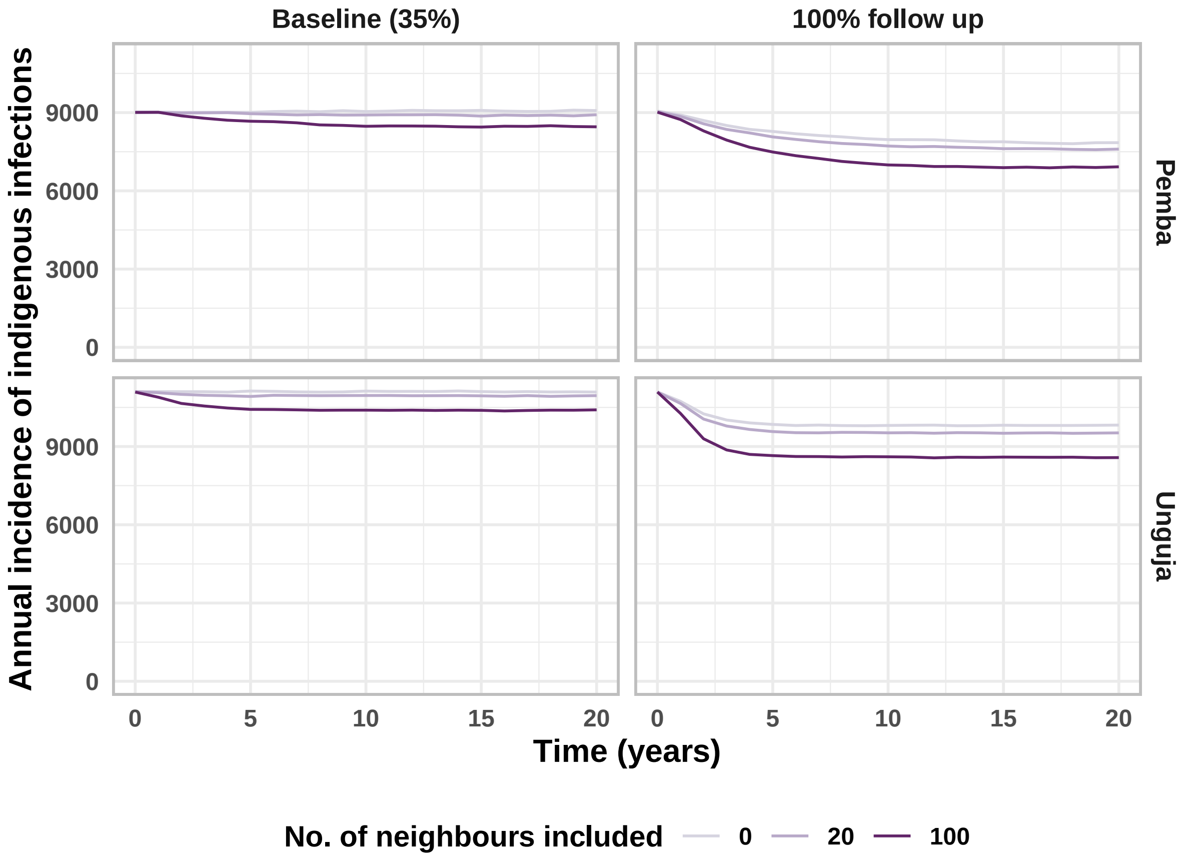

Figure S8: Median annual incidence of infections comparing the current reactive case detection (RCD) system to a system where 100% of malaria cases diagnosed at a health facility are followed up at the index household level and a range of number of neighbours are included in RCD.

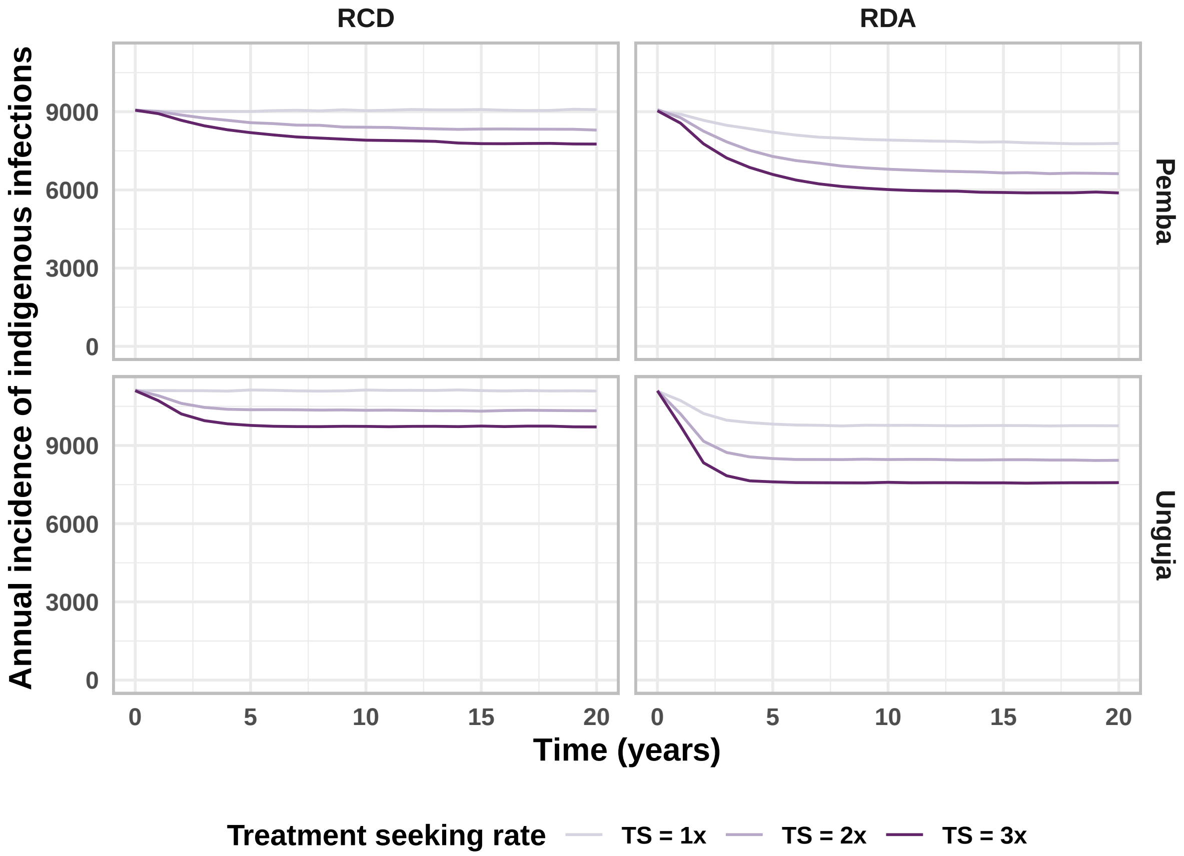

Figure S9: Median annual incidence of infections comparing the current reactive case detection (RCD) system to reactive drug administration (RDA; in reaction to detecting a case at a health facility, upon follow up, testing is skipped and antimalarials are given to all index household members) and increases in the rate at which people seek treatment (treatment seeking is abbreviated as 'TS').

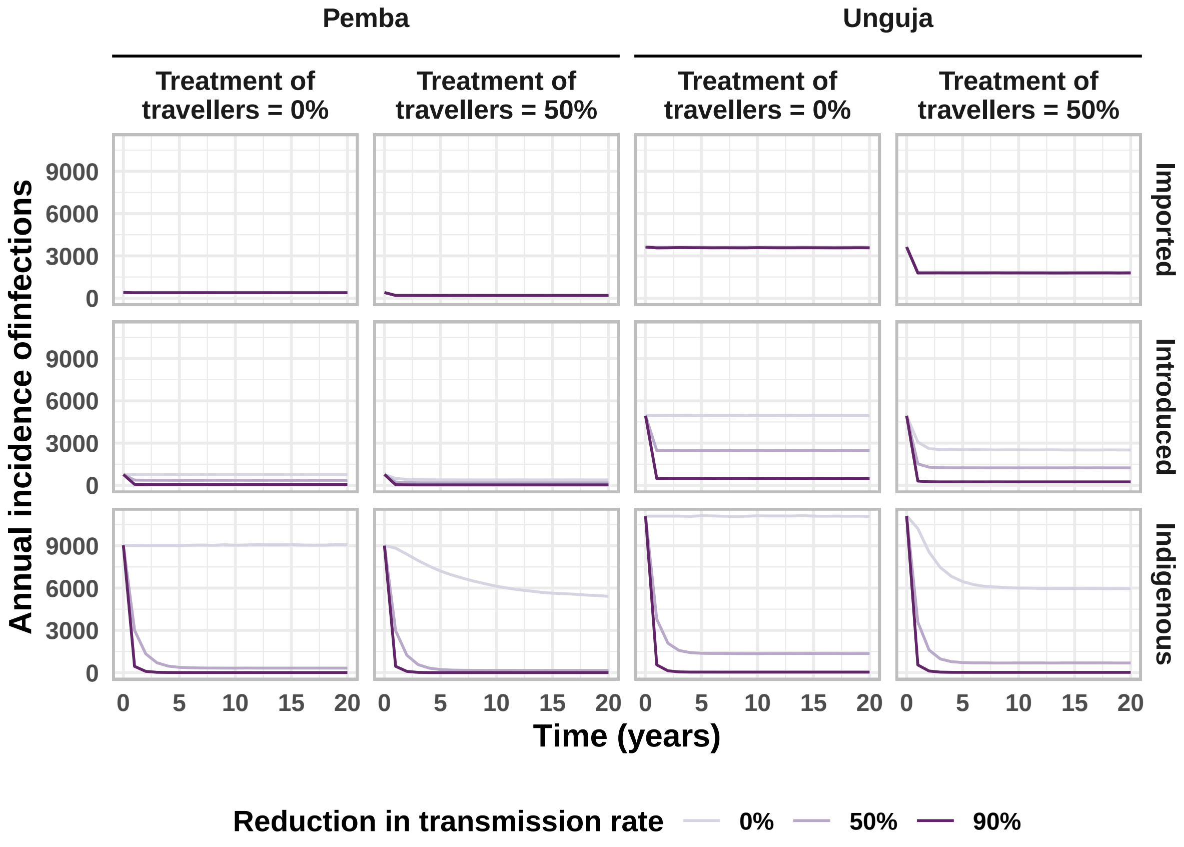

Figure S10: Median annual incidence of indigenous infections comparing the baseline interventions (reactive case detection for 35% of cases arriving at a health facility at the index household level only) to also treating a range of proportions of infected travellers, as well as vector control to reduce the malaria transmission rate on Zanzibar.

S3.2 Figures with all previously introduced interventions also in place at maximum values

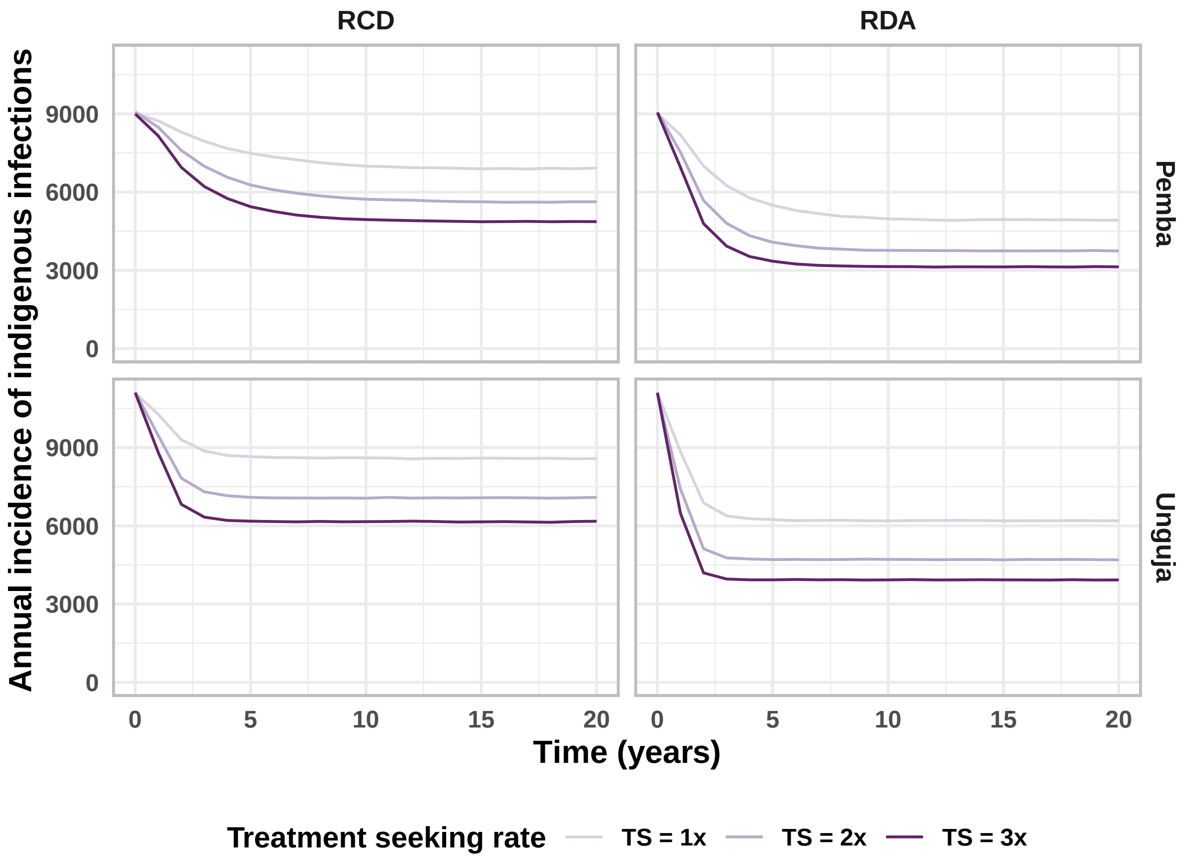

Figure S11: Median incidence of infections from 500 stochastic simulations comparing the current reactive case detection (RCD) system with 100% follow up of cases and 100 neighbours being included in testing and treating to reactive drug administration (RDA; in reaction to detecting a case at a health facility, upon follow up, testing is skipped and antimalarials are given to all index household members and 100 neighbours) and increases in the rate at which people seek treatment (treatment seeking is abbreviated as 'TS').

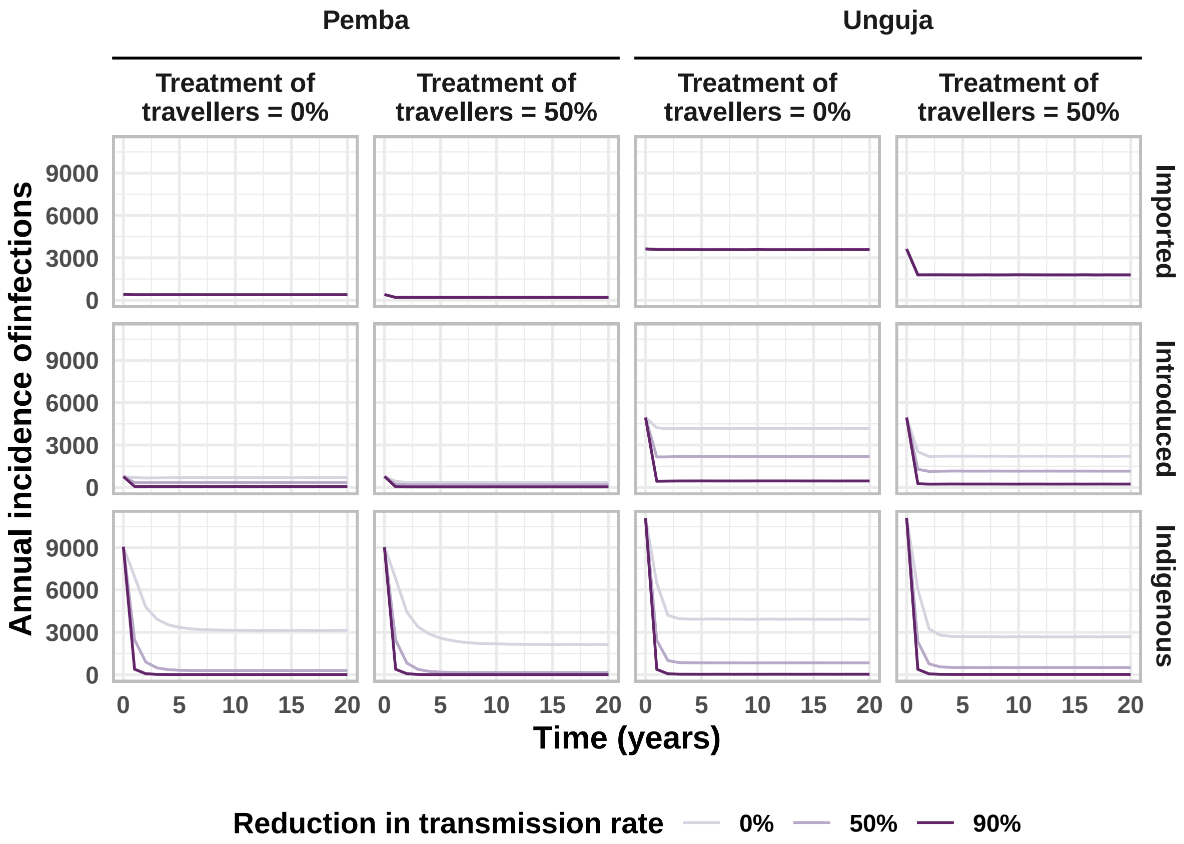

Figure S12: Median incidence of infections from 500 stochastic simulations comparing the impact of treating imported cases and reducing the malaria transmission rate on Zanzibar (both Pemba and Unguja). Here, we assume all reactive case detection-related interventions (100% follow up of all cases, treatment of the index household and 100 neighbours included in reactive drug administration, treatment seeking rate increased to 3 times the baseline rate) are also in effect.

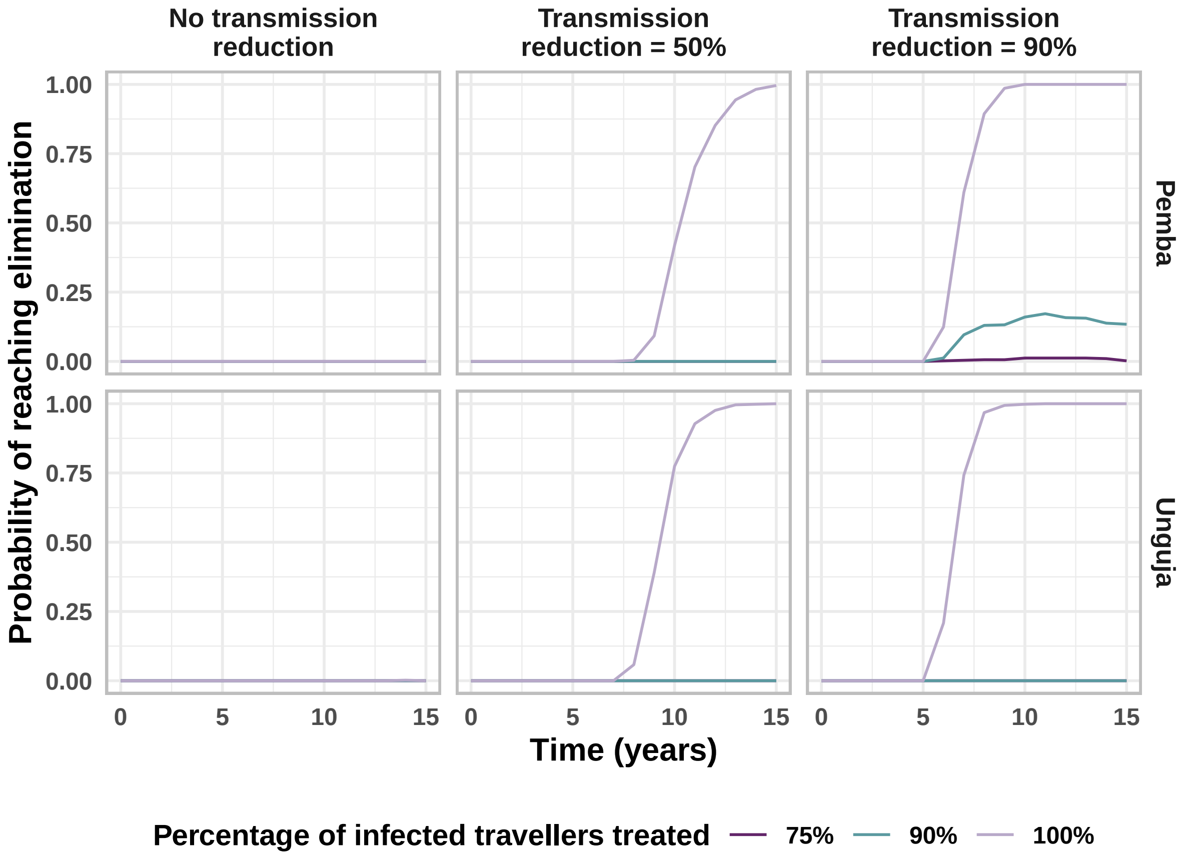

Figure S13: Proportion of stochastic simulations reaching elimination (three years with zero indigenous cases), starting from the introduction of interventions. We assume that the maximum values of all reactive case detection-related interventions (100% follow up of all cases, treatment of the index household and 100 neighbours included in reactive drug administration, treatment seeking rate increased to 3 times the baseline rate) are present and then simulate reducing the malaria transmission rate and treating a proportion of cases imported from mainland Tanzania.

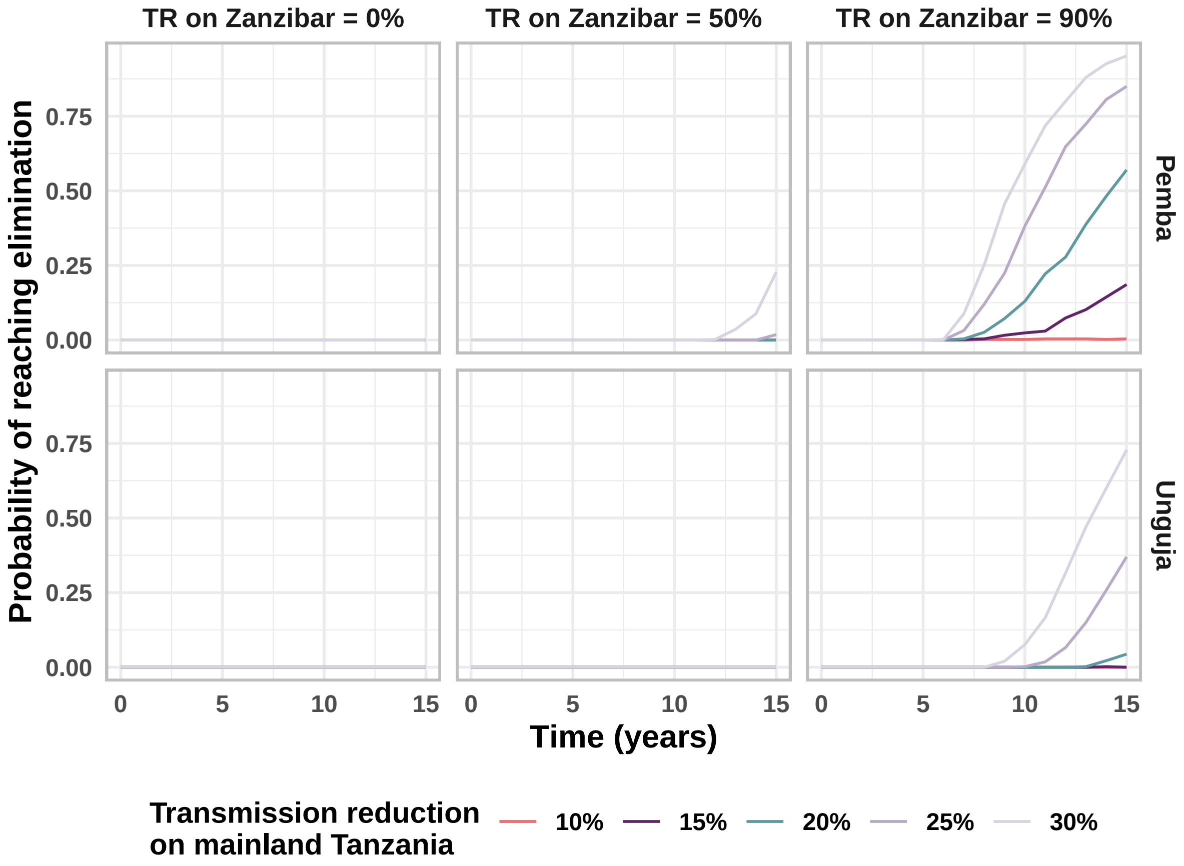

Figure S14: Proportion of stochastic simulations reaching elimination (three years with zero indigenous cases), starting from the introduction of interventions. We assume that the maximum values of all reactive case detection-related interventions (100% follow up of all cases, treatment of the index household and 100 neighbours included in reactive drug administration, treatment seeking rate increased to 3 times the baseline rate) are present and then simulate reducing the malaria transmission rate on both Zanzibar and mainland Tanzania. TR:transmission reduction.

S3.3 Probability of elimination over a longer period of time with 100% treatment of travellers

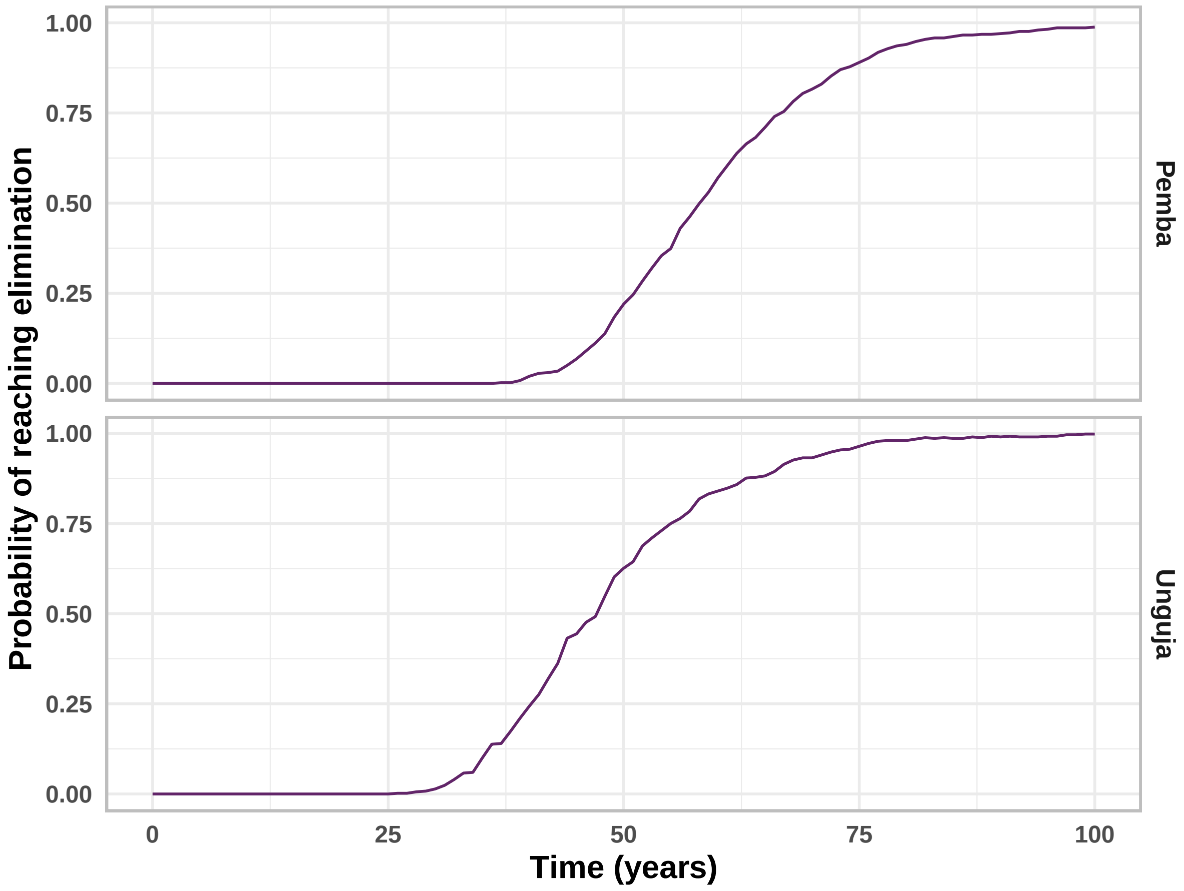

Figure S15: Proportion of stochastic simulations reaching elimination (three years with zero indigenous cases) when 100% of infected travellers from mainland Tanzania are treated, starting from year 0. We assume that all other interventions are at baseline values (reactive case detection for 35% of cases arriving at a health facility at the index household level only).

S4 Additional tables

	Number of imported cases	Number of infected visitors
**Pemba**	216	234
**Unguja**	1888	1829

Table S4: Median number of imported cases and infected visitors present on each patch at equilibrium.

References

[1] Tina van der Horst, Abdul-Wahid Al-mafazy, et al. Operational coverage and timeliness of reactive case detection for malaria elimination in Zanzibar, Tanzania. *The American Journal of Tropical Medicine and Hygiene*, 102(2):298–306, 2020.

[2] Aatreyee M Das, Manuel W Hetzel, et al. The impact of human mobility and reactive case detection on malaria transmission in Zanzibar. *medRxiv*, 2022.

[3] David L Smith and F Ellis McKenzie. Statics and dynamics of malaria infection in Anopheles mosquitoes. *Malaria Journal*, 3(1):1–14, 2004.

[4] Logan Stuck, Bakar S Fakih, et al. Malaria infection prevalence and sensitivity of reactive case detection in Zanzibar. *International Journal of Infectious Diseases*, 97:337 – 346, 2020.

[5] Simon I Hay and Robert W Snow. The Malaria Atlas Project: developing global maps of malaria risk. *PLOS Medicine*, 3(12):1–5, 2006.

[6] Tanzania National Bureau of Statistics. Population and housing census: population distribution by administrative areas. *Ministry of Finance, Dar es Salaam*, 2012.

[7] Sandip Mandal, Ram Rup Sarkar, et al. Mathematical models of malaria–a review. *Malaria Journal*, 10:202–202, 2011.

[8] Geoffrey M Jeffery and Don E Eyles. Infectivity to mosquitoes of *Plasmodium falciparum* as related to gametocyte density and duration of infection. *The American Journal of Tropical Medicine and Hygiene*, 4(5):781–789, 1955.

[9] Andrea Saltelli, Paola Annoni, et al. Variance based sensitivity analysis of model output. design and estimator for the total sensitivity index. *Computer Physics Communications*, 181(2):259–270, 2010.

[10] Jon Herman and Will Usher. Salib: An open-source python library for sensitivity analysis. *Journal of Open Source Software*, 2(9):97, 2017.

[11] W Sama, K Dietz, et al. Distribution of survival times of deliberate *Plasmodium falciparum* infections in tertiary syphilis patients. *Transactions of the Royal Society of Tropical Medicine and Hygiene*, 100(9):811–816, 2006.

[12] Arnaud Le Menach, Andrew J Tatem, et al. Travel risk, malaria importation and malaria transmission in Zanzibar. *Scientific Reports*, 1:93–93, 2011.

[13] Nakul Chitnis, Peter Pemberton-Ross, et al. Theory of reactive interventions in the elimination and control of malaria. *Malaria Journal*, 18(1):266, 2019.

[14] World Health Organization. *A framework for malaria elimination.* World Health Organization, 2017.
