# Supplementary material for: Modelling the impact of interventions on imported, introduced and indigenous malaria infections in Zanzibar, Tanzania
Source: Nat Commun. 2023 May 12;14:2750. doi: 10.1038/s41467-023-38379-8 (PMC10182017; doi:10.1038/s41467-023-38379-8)
Supplement: Supplementary file 3 — Reporting Summary [file 41467_2023_38379_MOESM3_ESM.pdf]

nature portfolio

Corresponding author(s): Aatreyee Mimi Das

Last updated by author(s): Mar 29, 2023

Reporting Summary

Nature Portfolio wishes to improve the reproducibility of the work that we publish. This form provides structure for consistency and transparency in reporting. For further information on Nature Portfolio policies, see our Editorial Policies and the Editorial Policy Checklist.

Statistics

For all statistical analyses, confirm that the following items are present in the figure legend, table legend, main text, or Methods section.

n/a	Confirmed	
☐	☒	The exact sample size (*n*) for each experimental group/condition, given as a discrete number and unit of measurement
☒	☐	A statement on whether measurements were taken from distinct samples or whether the same sample was measured repeatedly
☒	☐	The statistical test(s) used AND whether they are one- or two-sided<br>*Only common tests should be described solely by name; describe more complex techniques in the Methods section.*
☒	☐	A description of all covariates tested
☒	☐	A description of any assumptions or corrections, such as tests of normality and adjustment for multiple comparisons
☐	☒	A full description of the statistical parameters including central tendency (e.g. means) or other basic estimates (e.g. regression coefficient) AND variation (e.g. standard deviation) or associated estimates of uncertainty (e.g. confidence intervals)
☒	☐	For null hypothesis testing, the test statistic (e.g. F, t, r) with confidence intervals, effect sizes, degrees of freedom and P value noted<br>*Give P values as exact values whenever suitable.*
☒	☐	For Bayesian analysis, information on the choice of priors and Markov chain Monte Carlo settings
☒	☐	For hierarchical and complex designs, identification of the appropriate level for tests and full reporting of outcomes
☒	☐	Estimates of effect sizes (e.g. Cohen's *d*, Pearson's *r*), indicating how they were calculated

Our web collection on statistics for biologists contains articles on many of the points above.

Software and code

Policy information about availability of computer code

Data collection	No data was collected specifically for this study, therefore no software was used for data collection in this study.
Data analysis	The model, post-processing and plotting code can be found at https://github.com/SwissTPH/ZanzibarI3. Modelling, data analysis and plotting were conducted using Python version 3.6.6, numba version 0.39.0, R version 4.1.2 and ggplot2 version 3.3.5.

For manuscripts utilizing custom algorithms or software that are central to the research but not yet described in published literature, software must be made available to editors and reviewers. We strongly encourage code deposition in a community repository (e.g. GitHub). See the Nature Portfolio guidelines for submitting code & software for further information.

Data

Policy information about availability of data

All manuscripts must include a data availability statement. This statement should provide the following information, where applicable:

- Accession codes, unique identifiers, or web links for publicly available datasets
- A description of any restrictions on data availability
- For clinical datasets or third party data, please ensure that the statement adheres to our policy

The data required to run this model can be found at https://github.com/SwissTPH/ZanzibarI3. Publicly available data from the Malaria Atlas Project was also used to parameterise the model. This data can be found at https://data.malariaatlas.org.

Human research participants

Policy information about studies involving human research participants and Sex and Gender in Research.

Reporting on sex and gender	We use secondary data for this analysis. Primary data collection was reported in Stuck et al. International Journal of Infectious Diseases 2020; 97: 337–346.
Population characteristics	A proportion of the human research participants studied had a malaria diagnosis.
Recruitment	Recruitment occurred through the follow up of malaria cases diagnosed at health facilities and requesting consent at the households of symptomatic malaria patients. Details can be found in Stuck et al. International Journal of Infectious Diseases 2020; 97: 337–346.
Ethics oversight	Ethical approval for the collection of the data used in this study was obtained from the Zanzibar Medical Research Ethics Committee, the Institutional Review Boards of Tulane University and of the Ifakara Health Institute as well as the Ethics Commission of North-western and Central Switzerland.

Note that full information on the approval of the study protocol must also be provided in the manuscript.

Field-specific reporting

Please select the one below that is the best fit for your research. If you are not sure, read the appropriate sections before making your selection.

☒ Life sciences ☐ Behavioural & social sciences ☐ Ecological, evolutionary & environmental sciences

For a reference copy of the document with all sections, see nature.com/documents/nr-reporting-summary-flat.pdf

Life sciences study design

All studies must disclose on these points even when the disclosure is negative.

Sample size	500 computer simulations were run per intervention scenario to ensure ensure an adequate number of simulations to capture stochasticity in the system while ensuring short run times for each scenario.
Data exclusions	No data was excluded in this study.
Replication	As this was a computational study, experimental replicates do not apply
Randomization	As this was a computational study, randomization in the experiment does not apply.
Blinding	As this was a computational study, blinding was not relevant.

Reporting for specific materials, systems and methods

We require information from authors about some types of materials, experimental systems and methods used in many studies. Here, indicate whether each material, system or method listed is relevant to your study. If you are not sure if a list item applies to your research, read the appropriate section before selecting a response.

Materials & experimental systems

n/a	Involved in the study	
☒	☐	Antibodies
☒	☐	Eukaryotic cell lines
☒	☐	Palaeontology and archaeology
☒	☐	Animals and other organisms
☒	☐	Clinical data
☒	☐	Dual use research of concern

Methods

n/a	Involved in the study	
☒	☐	ChIP-seq
☒	☐	Flow cytometry
☒	☐	MRI-based neuroimaging
